# Vibration Sensor-Based Rotor Unbalance Identification for Micromotors Using LSTM Neural Networks

**DOI:** 10.3390/s26217053

**Published:** 2026-11-08

**Authors:** Xiaochen Hang, Huachong Fan, Dong Jiang

**Affiliations:** School of Mechanical and Electronic Engineering, Nanjing Forestry University, Nanjing 210037, China; fhc001@njfu.edu.cn (H.F.); jiangdong@njfu.edu.cn (D.J.)

**Keywords:** micromotor rotor, unbalance identification, LSTM neural network, rotor balancing

## Abstract

**Highlights:**

**What are the main findings?**

• An LSTM-based method directly identifies unbalance mass and phase on both correction planes of micromotor rotors from a single constant-speed vibration measurement, achieving a relative identification error below 5% for masses above 40 mg.
• Experimental balancing on three-slot and five-slot micromotor rotors demonstrates that the residual unbalance is reduced by 10.8–17.4% compared with the average influence coefficient method.

**What are the implications of the main findings?**

• The proposed method eliminates the need for trial weights and repeated start–stop cycles, improving detection efficiency for micromotor balancing.

**Abstract:**

To overcome the low accuracy and inefficiency of the conventional influence coefficient method for micromotor rotor balancing, this paper proposes a rotor unbalance identification approach based on a long short-term memory (LSTM) neural network. Synthetic vibration responses generated from the rotor-support dynamic model are used to pre-train the network, which is then fine-tuned with measured data, so that the knowledge learned from the idealized model is adapted to real measurement conditions. After min–max normalization, the data are fed into a multi-layer LSTM network that directly predicts the unbalance masses and phases on both correction planes from a single constant-speed vibration measurement. Simulation results show that the identification error remains below 5% for unbalance masses above 40 mg and within 4 mg for smaller masses. Experimental validation on three-slot and five-slot micromotor rotors demonstrates that the residual unbalance after correction is reduced by 10.8–17.4% depending on rotor type and correction plane, corresponding to an overall reduction of 15.4% in total residual unbalance mass. The proposed method eliminates repeated start–stop and trial-weight operations, significantly improving balancing efficiency. This work provides an effective approach for high-precision and rapid balancing of micromotor rotors.

## 1. Introduction

Micromotors are widely used in precision instruments, automotive systems, and industrial automation. Rotor unbalance, arising from material defects, machining tolerances, assembly errors, and thermal deformation during operation, is a primary cause of excessive vibration and premature failure [1,2]. Due to their compact and lightweight nature, micromotor rotors have extremely stringent unbalance tolerances, necessitating high-precision identification of unbalance mass and phase prior to correction [3,4]. In practice, displacement and Hall sensors are typically employed to capture the vibration amplitude and phase reference signals of the rotor-support system. The accuracy and efficiency of subsequent signal processing directly determine the achievable balancing precision.

The two most widely adopted traditional balancing methods are the influence coefficient method and the modal balancing method [5]. The former determines unbalance by establishing an influence coefficient matrix between vibration responses and known trial weights. Although studies [6,7,8,9] have refined its computational and experimental procedures, these approaches inherently require multiple start–stop cycles and trial-weight installations, which reduce detection efficiency and increase susceptibility to calibration errors. To address these limitations, researchers have explored transient and modal-based strategies, primarily for large flexible rotors. For instance, Zhao et al. [10,11,12] proposed a transient balancing method combined with dynamic load identification (DLI) to estimate unbalance parameters in general rotor systems. However, such methods rely on transient startup/shutdown processes of large flexible rotors and are not directly applicable to the constant-speed rigid rotors of micromotors. Li et al. [13] developed a V-shaped milling correction method based on a discrete vector model to improve balancing accuracy for micromotor armatures. While effective, its correction precision is limited by the mechanical resolution of the milling process, particularly for extremely small unbalance magnitudes. More recently, trial-weight-free balancing has been pursued through augmented Kalman filtering [14] and unsupervised deep Lagrangian networks [15], although both target flexible rotors. For micromotor rotors, our recent work proposed a two-step weight-removing strategy based on a discrete vector model [16], which copes with large initial unbalance. However, weight removal addresses only the correction step, and the achievable accuracy remains bounded by the precision of the preceding unbalance identification.

These limitations have motivated the adoption of data-driven approaches that directly learn the mapping from vibration responses to unbalance parameters. Recently, machine learning and deep learning have been increasingly applied to rotor unbalance analysis and fault diagnosis [17,18]. Physics-informed neural networks have been used to estimate unbalance-related parameters of rotating shafts directly from displacement signals [19]; an LSTM network trained on a combination of simulated and measured responses has been used to recognize unbalanced positions in rotor systems [20]; and transfer learning has been established as an effective means of bridging simulated and measured data in rotating machinery diagnosis [21]. Gohari et al. [22] extracted time- and frequency-domain features from bearing acceleration signals to estimate unbalance parameters using artificial neural networks; in follow-up work, they applied K-nearest neighbor and decision tree algorithms to multi-disk rotors [23]. Nevertheless, these methods rely heavily on handcrafted features, which may limit their generalization across different rotor configurations. More critically, most existing data-driven studies focus on fault classification or signal prediction, rather than quantitatively identifying the unbalance mass and phase on specific correction planes [24]. Dong et al. [25] combined an LSTM network with zero-phase filtering to process unbalance signals of micromotor rotors. However, their study focused on signal reconstruction and feature extraction rather than quantitative identification of unbalance mass and phase.

To address this gap, this paper proposes an LSTM-based method for direct identification of unbalance mass and phase on both correction planes of micromotor rotors. Specifically, an LSTM network is designed to map vibration responses and geometric parameters to the unbalance masses and phases on the corresponding correction planes, thereby eliminating the need for trial weights and repeated start–stop operations. Experimental validation is conducted on three-slot and five-slot micromotor rotors, and the proposed method is compared against the average influence coefficient (AIC) method. Experimental results demonstrate a 10.8–17.4% reduction in residual unbalance and a significant improvement in detection efficiency compared with the AIC method.

## 2. Rotor Unbalance Identification Method Based on LSTM Neural Network

### 2.1. Equations of Motion for Rotor Unbalance

An accurate dynamic model of the rotor-support system is essential for analyzing the relationship between unbalance parameters and vibration responses.

**Figure 1:**
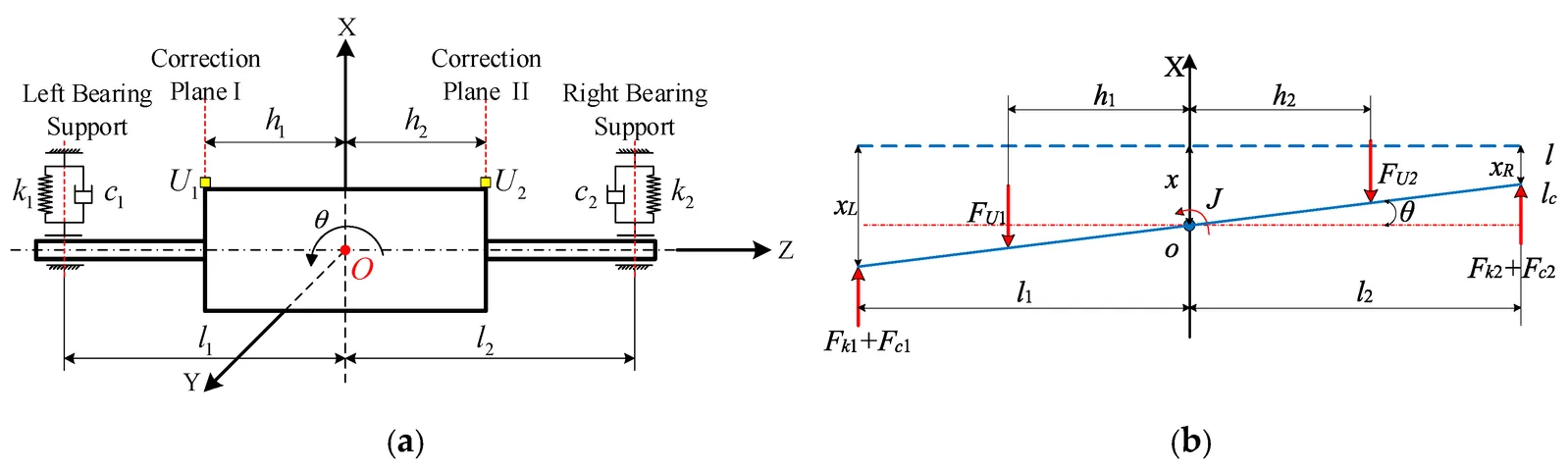
Schematic diagram of the rotor dynamic balancing tester system. (**a**) Dynamic model of the rotor-support system; (**b**) force diagram of the system during rotor vibration.

Figure 1 illustrates the rotor dynamic balancing test system. Under V-shaped supports, a displacement sensor is positioned horizontally to measure the rotor vibration in the horizontal direction, while vertical vibration is neglected. The sensor is connected to a spring leaf via a movable rod, and the damping on the spring leaf must be taken into account. Micromotor rotors, characterized by small mass and short shaft length, are treated as rigid rotors. Consequently, the system can be simplified as a damped two-degree-of-freedom spring-mass system, whose dynamic model is depicted in Figure 1a.

The rotor centroid O is set as the coordinate origin, with the rotation axis defined as the Z-axis and the X-axis perpendicular to the Z-axis in the horizontal plane. The rotor rotates about the Z-axis at an angular velocity ω. The rotor mass is denoted as *m*, and its moment of inertia about the Y-axis is *J*. The stiffness and damping coefficients of the springs at the left and right supports are (*k*_1_, *k*_2_) and (*c*_1_, *c*_2_), respectively. The distances from the left and right supports to the centroid O are *l*_1_ and *l*_2_. The correction planes I and II are located at distances *h_i_* (*i* = 1, 2) from the centroid O. The unbalance vectors *U_i_* (*i* = 1, 2) on correction planes I and II are expressed as

(1) *U_i_* = *m_i_r_i_*

where *m_i_* denotes the unbalance mass and *r_i_* denotes the position vector of the unbalance mass on the correction plane, including its initial phase angle *φ_i_* (*i* = 1, 2).

Let x represent the translational displacement of the centroid O along the X-axis, and *θ* represent the rotational displacement of the rotor about the Y-axis. The displacements at the left and right supports, denoted as *x_L_* and *x_R_*, are given by

(2) $$\left\{\begin{array}{c} x_{L}=x-l_{1}\theta \\ x_{R}=x+l_{2}\theta \end{array}\right.$$

When the rotor vibrates on its supports, a force analysis of the system is performed, as shown in Figure 1b. Line *l* denotes the rotor axis at rest, while line *lc* denotes the rotor axis during unbalanced vibration. At a given instant during rotation, *F_U_*_1_ and *F_U_*_2_ are the centrifugal inertial forces caused by the left and right unbalances; *F_k_*_1_ and *F_k_*_2_ are the spring forces at the left and right supports; and *Fc*_1_ and *Fc*_2_ are the corresponding damping forces.

Based on force and moment balance, and accounting for the additional mass of the rotor and supports, the differential equations of motion for the rotor-support system are

(3) $$\left\{\begin{array}{c} m^{*}\ddot{x}+c_{1}{\dot{x}}_{L}+c_{2}{\dot{x}}_{R}+k_{1}x_{L}+k_{2}x_{R}=\sum_{i=1}^{2}U_{i}\omega^{2}\cos\left(\mathrm{\omega t}+\varphi_{i}\right)\\ J\ddot{\theta }-c_{1}{\dot{x}}_{L}l_{1}+c_{2}{\dot{x}}_{R}l_{2}-k_{1}x_{L}l_{1}+k_{2}x_{R}l_{2}=-h_{1}U_{1}\omega^{2}\cos\left(\mathrm{\omega t}+\varphi_{1}\right)+h_{2}U_{2}\omega^{2}\cos\left(\mathrm{\omega t}+\varphi_{2}\right) \end{array}\right.$$

Substituting Equations (1) and (2) into Equation (3) yields the matrix form of the equations of motion:

(4) $$\begin{array}{l} \left[\begin{array}{cc} m^{*} & 0\\ 0 & J \end{array}\right]\left[\begin{array}{c} \ddot{x}\\ \ddot{\theta } \end{array}\right]+\left[\begin{array}{cc} c_{1}+c_{2} & -c_{1}l_{1}+c_{2}l_{2}\\ -c_{1}l_{1}+c_{2}l_{2} & c_{1}l_{1}^{2}+c_{2}l_{2}^{2} \end{array}\right]\left[\begin{array}{c} \dot{x}\\ \dot{\theta } \end{array}\right]+\left[\begin{array}{cc} k_{1}+k_{2} & -k_{1}l_{1}+k_{2}l_{2}\\ -k_{1}l_{1}+k_{2}l_{2} & k_{1}l_{1}^{2}+k_{2}l_{2}^{2} \end{array}\right]\left[\begin{array}{c} x\\ \theta \end{array}\right]\\ =\left[\begin{array}{c} \sum_{i=1}^{2}m_{i}r_{i}\omega^{2}\cos\left(\mathrm{\omega t}+\varphi_{i}\right)\\ -h_{1}m_{1}r_{1}\omega^{2}\cos\left(\mathrm{\omega t}+\varphi_{1}\right)+h_{2}m_{2}r_{2}\omega^{2}\cos\left(\mathrm{\omega t}+\varphi_{2}\right) \end{array}\right] \end{array}$$

which can be expressed in compact form as

(5) $$M\ddot{X}+C\dot{X}+\mathrm{KX}=F$$

where

***X***—the system vibration response vector;

***M***—the system mass matrix, where the total vibrating mass m* is m* = (*m* + *m*_0_) with *m* being the rotor mass and *m*_0_ the additional support mass, and the moment of inertia is *J*;

***C***—the system damping matrix, with damping applied to the springs at both supports;

***K***—the system stiffness matrix;

***F***—the external excitation matrix.

### 2.2. LSTM Neural Network Architecture

**Figure 2:**
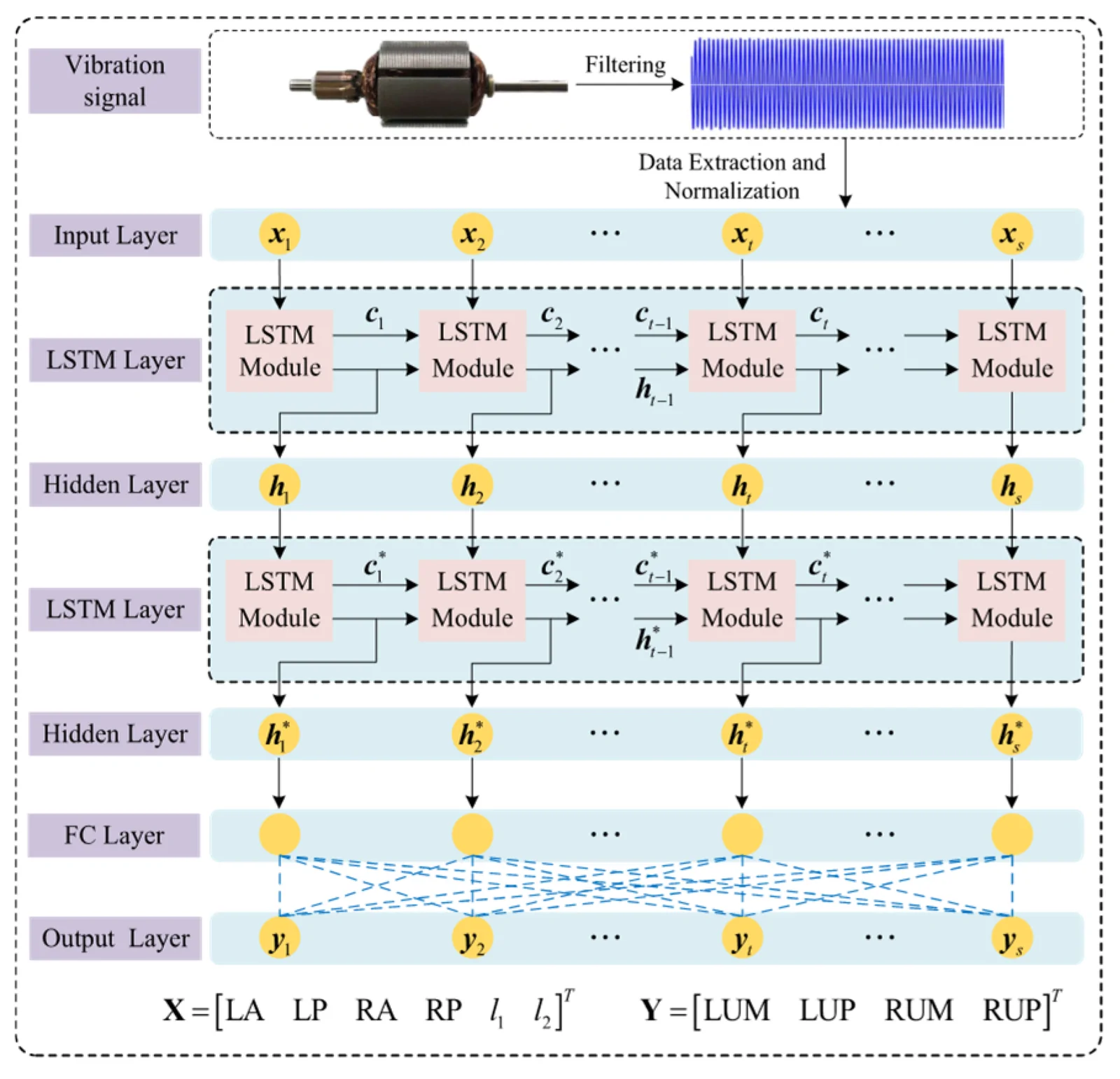
Neural network architecture for rotor unbalance identification.

Rotor unbalance identification constitutes a multi-input multi-output regression problem. A multi-layer regression LSTM neural network model is therefore constructed. The network architecture, shown in Figure 2, comprises an input layer, two LSTM layers, two hidden layers, and a fully connected output layer.

**Input and Output Design**: The input layer receives six parameters: the vibration amplitudes and phases at the left and right measurement planes (LA, LP, RA, RP), along with the key geometric parameters (*l*_1_, *l*_2_) of the rotor-support system. The output layer produces four parameters *y_i_* (*i* = 1, 2…4). They are the unbalance masses and phases on the left and right correction planes Y = (LUM, LUP, RUM, RUP)^T^. To present the input vector X = [LA LP RA RP *l*_1_ *l*_2_]^T^ to the recurrent layer, its six components are mapped onto six time steps *x_i_* (*i* = 1, 2…6) with one feature per step. The time index *t* therefore enumerates the features of one sample and has no physical meaning as a sampling instant. The network does not exploit any temporal dependence of the vibration signal.

**Hidden Layer Design**: The network comprises two stacked LSTM layers with 200 hidden units each, followed by two fully connected hidden layers and a linear output layer of four nodes for the unbalance parameters. The number of nodes of the two fully connected hidden layers is determined from the empirical formula:

(6) $$n_{m}=\sqrt{n_{x}+n_{y}}+a$$

where *n_m_* is the number of hidden layer nodes, *n_x_* is the number of input layer nodes, *n_y_* is the number of output layer nodes, and *a* is a constant in the range [1,10]. Accordingly, the first and second fully connected hidden layers are set to 12 and eight nodes, respectively. The ReLU activation function is selected for these two hidden layers to enhance the nonlinear fitting capability and mitigate the vanishing gradient problem, and the output layer is linear for regression. The 200 hidden units of the LSTM layers denote the hidden-state dimension of the recurrent layers.

The network was implemented and trained in MATLAB R2023a Deep Learning Toolbox using the Adam optimizer with first-moment decay factor β_1_ = 0.9, to minimize the half mean-squared-error loss; the training settings are listed in Table 1.

### 2.3. Identification Workflow

**Figure 3:**
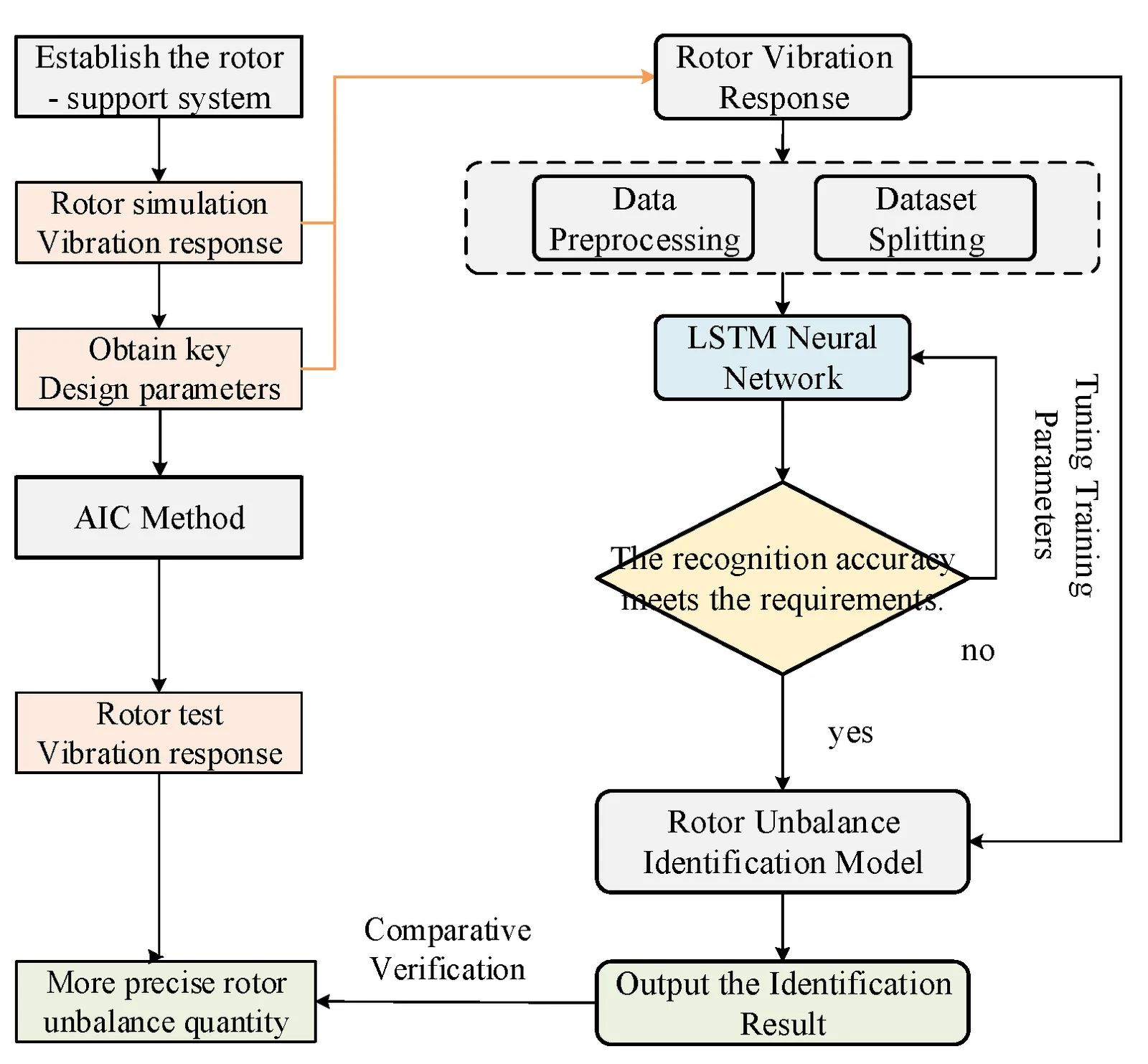
Workflow of the rotor unbalance identification.

As illustrated in Figure 3, the LSTM-based rotor unbalance identification follows the steps below:

(1) Design the network architecture and configure training parameters according to the input-output characteristics.
(2) Generate simulated vibration response signals by adding trial masses of varying magnitudes at different angular positions on the rotor model. The network is trained in two stages: pre-training on the simulated data, followed by fine-tuning on the experimental data; the two data sources are partitioned separately, as detailed in Section 3.1.
(3) Apply min–max normalization to the input data, with the normalization parameters computed exclusively from the training data, to improve convergence speed and training accuracy. The key design parameters *l*_1_ and *l*_2_ of the rotor-support dynamic model are incorporated as input variables for network training.
(4) Train the network and evaluate model performance, then use the trained model to identify rotor unbalance.

## 3. Case Study

### 3.1. Dataset Construction and Model Training

Training deep neural networks requires sufficient and diverse labeled data. However, for micromotor rotors, obtaining labeled experimental data is challenging. The unbalance masses of micromotor rotors are typically on the order of tens of milligrams, and performing a complete balancing calibration procedure for each labeled sample is extremely time-consuming. To overcome this data scarcity issue, a rotor-support system dynamic simulation model is employed to generate vibration response data. The simulated vibration responses were generated using the rotor-support dynamic model of Section 2.1. The baseline parameters are rotor mass m = 80 g, moment of inertia J = 1.6 × 10^−5^ kg·m^2^, rotor radius r = 23 mm, distances of the two correction planes from the mass centroid h_1_ = h_2_ = 15 mm, nominal support stiffness k_1_ = k_2_ = 30 kN/m, nominal damping coefficients c_1_ = c_2_ = 3 N·s/m, and additional support mass m_0_ = 20 g. The operating speed of rotors was 2160 r/min. For each sample, the unbalance on each correction plane was realized as a trial mass attached at a known angular position, and the steady state 1× vibration amplitudes and phases at the two measurement planes (LA, LP, RA, RP) were computed. The unbalance masses on planes I and II were sampled independently over 1–200 mg following a log-uniform distribution, which increases the sample density at small unbalances where identification is most difficult and covers the range of initial unbalances observed experimentally. The unbalance phases were sampled randomly over the 0–360° range, and the support distances *l*_1_ and *l*_2_ were sampled uniformly and independently over 26–34 mm. To enhance the robustness of the identification model to manufacturing variability and measurement noise, every sample was generated with the rotor mass perturbed independently by up to ±10% of nominal values, and with the operating speed perturbed by up to ±2%. In addition, zero-mean complex Gaussian noise with a standard deviation of 0.15 μm was superimposed on the simulated responses. Consequently, the dataset spans a wider range of dynamic characteristics than a single nominal rotor, and the identification accuracy reported already incorporates the effects of measurement noise and parameter uncertainty.

In addition, 80 sets of experimental data from our previous work [25,26] are used. The rotors from which these data were measured are entirely different individuals from the eight rotors used for the balancing validation in Section 3.2. Each data sample comprises six input parameters and four output parameters as mentioned before, with parameter definitions provided in Table 2. Representative training samples are presented in Table 3.

The vibration response data contain amplitude and phase quantities with different dimensions and widely varying value ranges, which can degrade training stability and convergence. Min–max normalization is therefore applied to map the data to the [0, 1] interval, eliminating dimensional effects and accelerating convergence. The normalized data are shown in Table 4.

A two-stage training strategy was adopted. In the pre-training stage, the 10,081 simulated samples were randomly divided into a pre-training set (90%, 9073 samples) and a pre-training validation set (10%, 1008 samples). In the fine-tuning stage, the 80 experimental samples were randomly divided into a fine-tuning training set (80%, 64 samples) and a fine-tuning validation set (20%, 16 samples). Fine-tuning started from the pre-trained weights and proceeded for at most 30 epochs at a learning rate of 1 × 10^−4^, with early stopping after five epochs without improvement on the fine-tuning validation set. All other settings followed Table 1. The reduced learning rate and early stopping were adopted to adapt the simulation-learned mapping to real measurements without overfitting the limited experimental data. Min–max normalization parameters were derived solely from the pre-training set and applied unchanged to all subsequent data.

**Figure 4:**
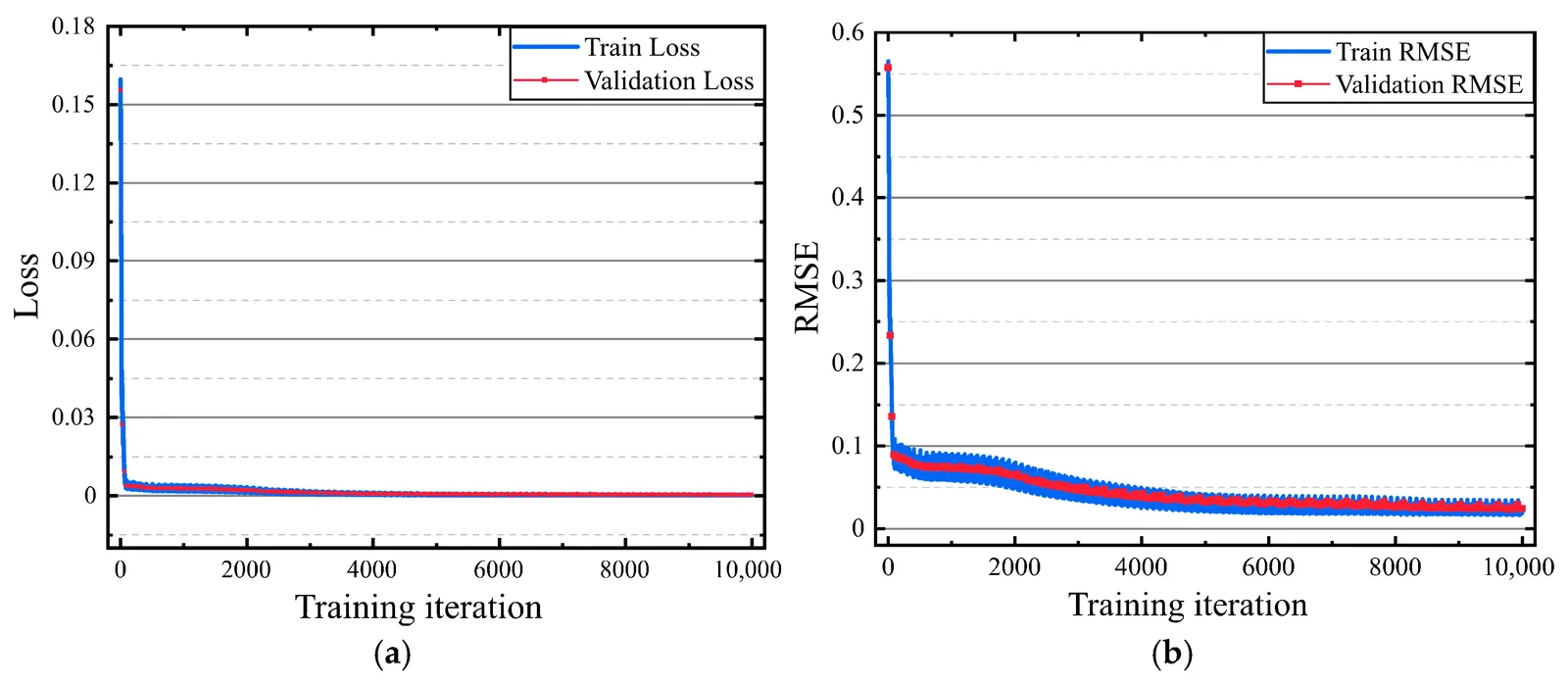
Training convergence of the LSTM network: (**a**) Loss; (**b**) RMSE.

**Figure 5:**
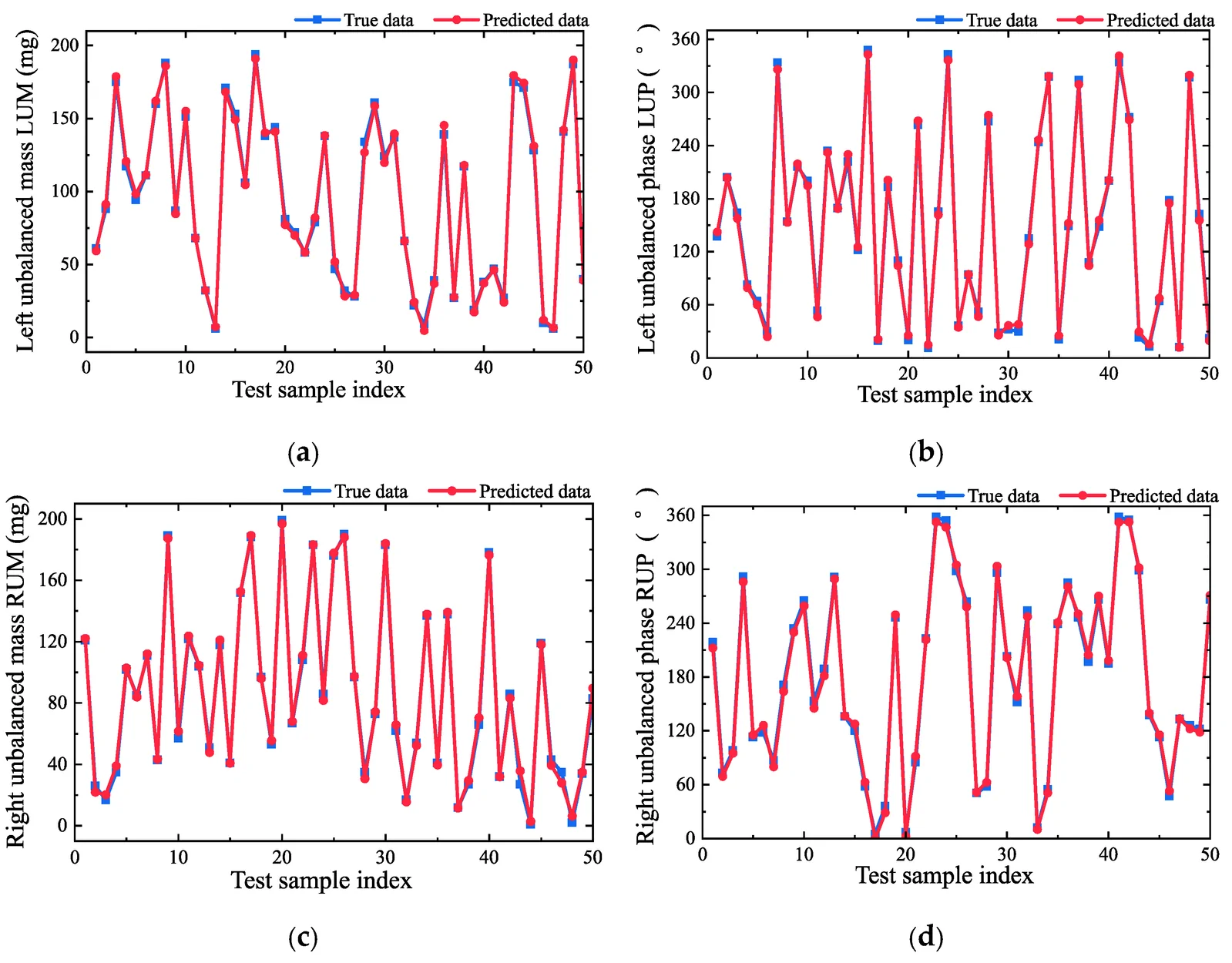
Rotor unbalance identification results using the trained model: (**a**) LUM; (**b**) LUP; (**c**) RUM; (**d**) RUP.

The constructed LSTM network was then trained. Because the number of training epochs (100) is smaller than the learn-rate drop period (200 epochs), the piecewise schedule was never triggered, and the learning rate remained constant at its initial value of 0.001 throughout training. The loss function, which quantifies the discrepancy between network outputs and target values, serves as a critical indicator of training performance. During each training iteration, the loss was computed and network weights and biases were updated accordingly, while the validation set was concurrently used to monitor generalization. The loss and root-mean-square error (RMSE) curves during training are presented in Figure 4. Both quantities are computed on the min–max-normalized targets. The RMSE is therefore dimensionless, and the mass and phase errors are combined with equal weights in normalized space. Both training and validation losses and RMSE values declined to near zero and converged steadily, indicating that the model performed well on both training and validation with no indication of overfitting. Fifty groups were randomly sampled from the pre-training validation set, which comprises 10% of the simulated data. The response data of these 50 groups were input into the trained model to predict the unbalance, and the predictions were compared with the 50 true unbalance values used in the simulation. The comparison results are shown in Figure 5. Among them, the identified values of five representative simulated samples are compared with the true values in Table 5. Both Figure 5 and Table 5 demonstrate the accuracy of the LSTM-based unbalance prediction model.

As shown in Table 5, for small unbalance masses, the relative identification error is larger, yet the absolute error consistently remains below 4 mg. For larger unbalance masses, the relative error is less than 5%. In practice, measurement noise, sensor nonlinearity, and environmental disturbances inevitably introduce additional errors, and the residual unbalance after correction may exceed the simulation-predicted values.

### 3.2. Experimental Validation

**Figure 6:**
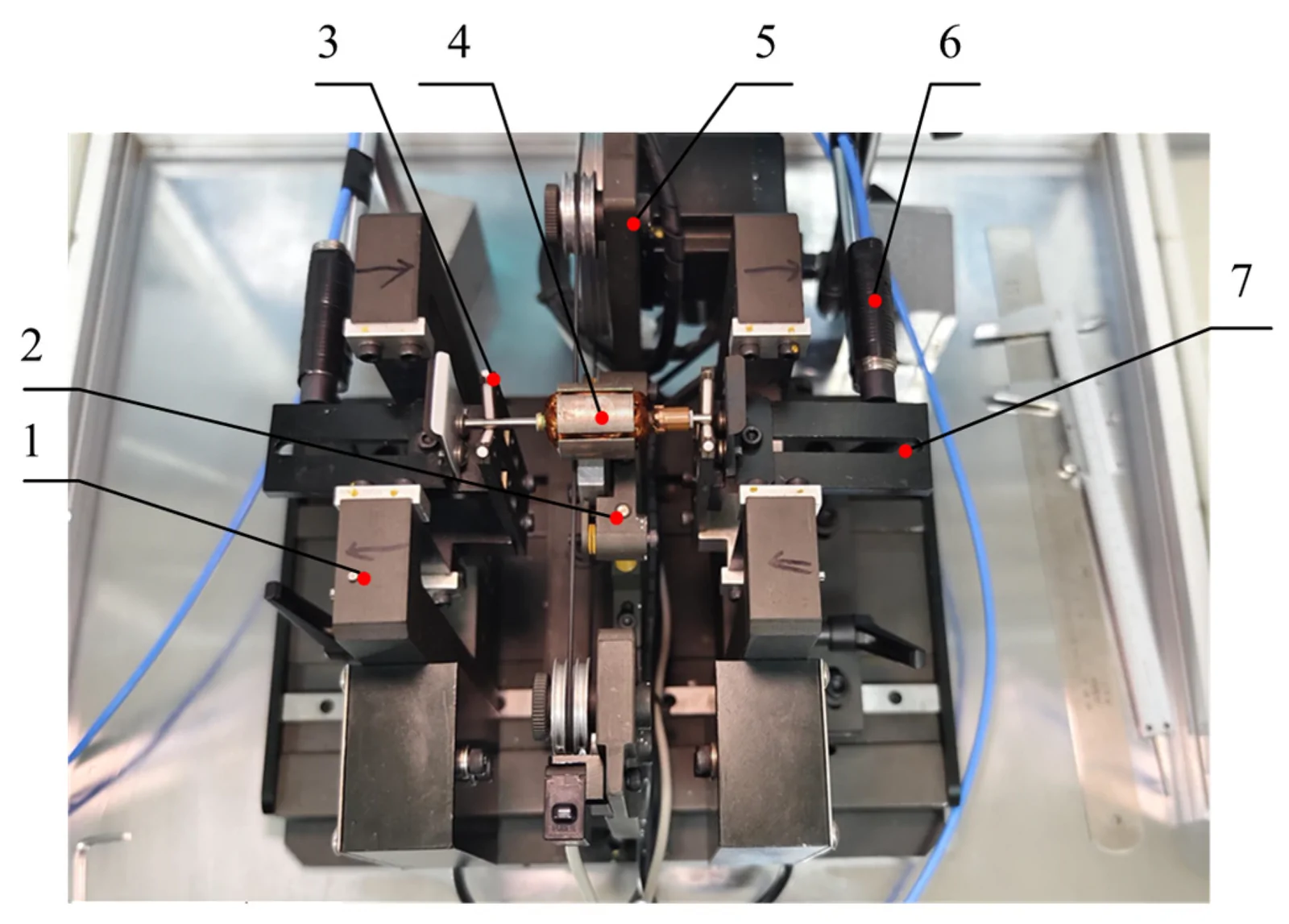
Experimental setup for dynamic balancing. (1. Calibration magnet; 2. Hall sensor; 3. V support; 4. Rotor; 5. Stepper motor; 6. Displacement sensor; 7. Swing frame).

The experimental platform is illustrated in Figure 6. It primarily consists of rotor supports, a stepper motor, a pulley-belt system, sensors, and a fixed base. Two supports, positioned on opposite sides of the base, accommodate the displacement sensors, while a Hall sensor is placed between the supports. The pulley is connected to the stepper motor via a belt. During unbalance signal acquisition, the rotor is placed on the supports, with its lower side in contact with the belt, and the stepper motor provides the driving torque for rotation. The displacement sensors measure the vibrations of the supports, which indirectly reflect the vibrations at the two correction planes, whereas the Hall sensor captures the rotor phase reference.

The displacement sensors are DONGHUA 5E102 (The equipment was sourced from Jiangsu Donghua Testing Technology Co., Ltd., Jingjiang, China.), with a range of ±2 mm, a repeatability of 0.05 μm, and a maximum sampling frequency of 10 kHz. The Hall sensor is a HUAXIN HX6571KSO (The equipment was sourced from Wuxi Huaxin Sheng Technology Co., Ltd., Wuxi, China), with a maximum sampling frequency of 69 kHz. The operating speed is 2160 rpm, the sampling frequency is 1 kHz, and 5 s of data are acquired after the system reaches steady state under each operating condition. Filtering was performed using the filtering algorithm built into the DONGHUA DH5922D signal acquisition unit to remove laboratory environmental noise.

Based on the vibration response data measured experimentally, the trained model outputs the initial unbalance of the rotor. Correction masses are then added according to the identified unbalance, and the residual unbalance is re-measured after balancing. For comparison, the AIC method was applied under identical experimental conditions.

Owing to inherent variations among individual rotors, the influence coefficients are taken as the average values obtained from multiple measurements, which helps to reduce scatter and improve identification accuracy. The procedure of the AIC method is as follows:

**Step 1. Initial run:** Run the rotor up to the balancing speed and record the initial vibration vectors (amplitude and phase) at each measurement plane.

**Step 2. Multi-position trial weighting:** Divide the rotor circumference into *K* equally spaced angular positions, e.g., *K* = 4 at 90° intervals. Attach a known trial weight of fixed magnitude successively at each of the *K* positions on the correction plane, and record the corresponding vibration vectors under the same speed.

**Step 3. Coefficient identification:** For each angular position *K*, calculate the influence coefficient α*^K^* as the complex ratio of the vibration change to the trial unbalance.

**Step 4. Averaging:** Average the *K* identified coefficients to obtain the average influence coefficient α_average_.

**Step 5. Matrix assembly and solution:** Repeat Steps 2–4 for every correction plane to build the average influence coefficient matrix, then solve the balancing equations for the correction masses and their angular positions.

**Step 6. Verification:** Apply the correction masses, re-run the rotor, and check the residual vibration.

**Figure 7:**
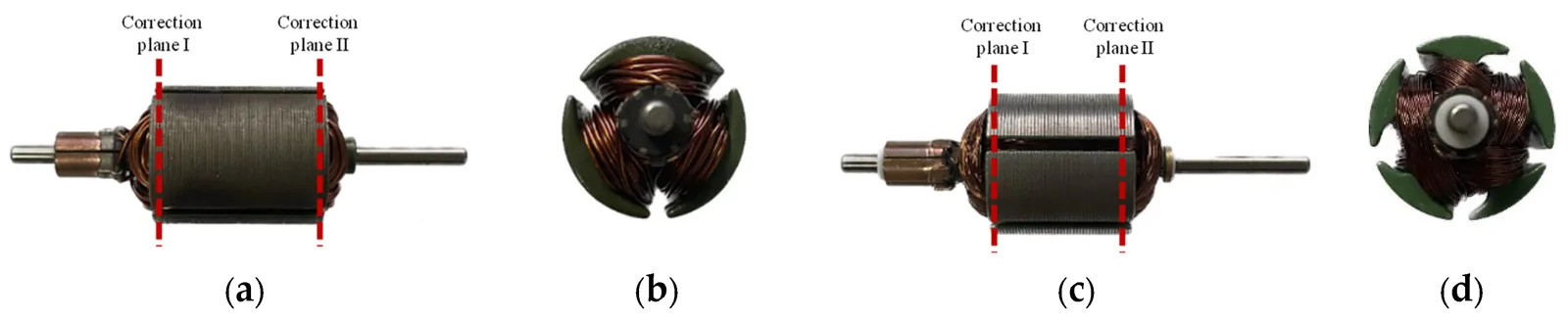
The three-slot rotor and five-slot rotor used in tests. (**a**) Radial view of three-slot rotor; (**b**) axial view of three-slot rotor; (**c**) radial view of five-slot rotor; (**d**) axial view of five-slot rotor.

Three-slot and five-slot micromotor rotors are shown in Figure 7. The masses of the three-slot rotor and the five-slot rotor are 80 g and 60 g, respectively, and both rotors have a radius of 23 mm. Four three-slot rotors were selected; vibration signals were acquired and initial unbalances on both the left and right sides were identified. Correction was then performed using the added-mass method, and residual unbalances were re-measured. The same procedure was followed for the five-slot rotors. Although the number of tested rotors is limited, the eight samples span a range of initial unbalance magnitudes (20–150 mg) and phase angles (33–338°), covering the typical operating envelope of micromotor rotors in production.

**Figure 8:**
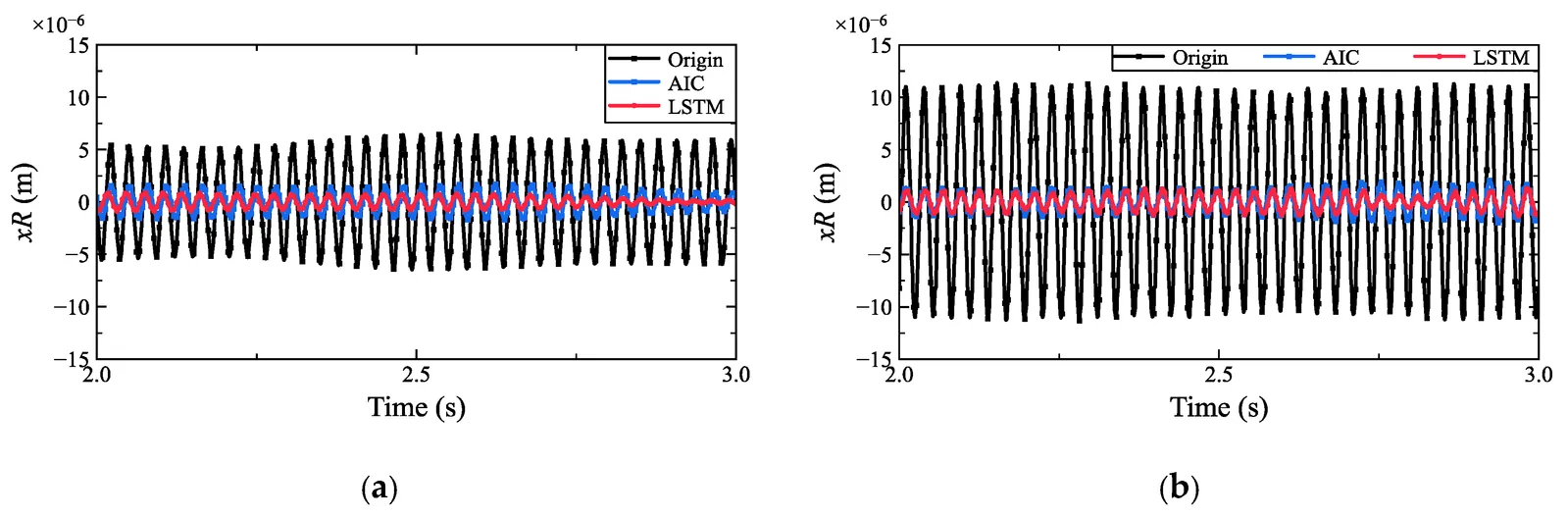
Vibration responses of the three-slot and five-slot rotors after balancing: (**a**) displacement xR at the right support of the three-slot rotor; (**b**) displacement xR at the right support of the five-slot rotor.

All balancing trials followed a fixed protocol identical for the two methods. All reported mass values are equivalent masses referred to the common correction radius r = 23 mm, and all angular quantities are measured in the direction of rotation from the trigger mark of the Hall sensor. The initial unbalance values in Table 6 were identified independently by the AIC and LSTM methods from the same baseline response, and the residual unbalance in Table 7 was identified by each method from the re-measured response after correction, while the residual vibration responses in Figure 8 provide a method-independent comparison. Since correction was performed by mass addition, the initial unbalance state was restored exactly by removing the added masses, and an ABBA crossover sequence was adopted. On one rotor the AIC method was applied first, and on another rotor the LSTM method was applied first, so that any systematic drift of the test rig over time would affect the two methods equally. The correction masses were weighed to better than 1 mg and placed radially within ±0.2 mm, giving a placement error bound of approximately 5% of the target correction that is common to both methods. Each 5 s steady-state record was filtered by the algorithm built into the DH5922D acquisition unit, and the amplitude and phase at each measurement plane (LA, LP, RA, RP) were extracted by the LSTM-ZPF algorithm, which was discussed in our previous work [25].

A comparison of unbalance identification results obtained by the average influence coefficient method and the proposed LSTM-based method is presented in Table 6. Mass deviation is calculated as **e***_m_* = avg. {|*m*_AIC_ − *m*_LSTM_|/*m*_LSTM_}. Phase deviation is calculated as **e***_φ_* = avg. {|*φ*_AIC_ − *φ*_LSTM_|}. As can be observed, the two methods yield highly consistent results, particularly when the initial unbalance masses on both correction planes exceed 40 mg, where the discrepancy remains within 4%. For smaller unbalance masses, the difference between the two methods can reach up to 4 mg, corresponding to a relative deviation of 17.3%.

For every one of the eight rotors, the total residual unbalance after LSTM balancing is lower than that obtained with the AIC method. The mean paired difference is 3.1 mg per rotor, with a standard deviation of 1.6 mg. A paired t-test confirms that this improvement is statistically significant: t (7) = 5.38, *p* = 0.001. The 95% confidence interval for the mean improvement spans 1.8 to 4.5 mg, and the effect size reaches d = 1.9. Since the sample size is small, the non-parametric Wilcoxon signed-rank test was also applied and leads to the same conclusion, with *p* = 0.008. The same picture emerges at the level of individual correction planes: among the 16 rotor–plane combinations, 13 show a reduced residual unbalance while none becomes worse. It is worth emphasizing that both methods process exactly the same measured data of each rotor. Measurement noise therefore affects the two methods in common mode and is largely canceled in the paired differences, so the observed improvement reflects a systematic difference between the two identification methods rather than fluctuations of the measurement or correction process.

Based on the identified unbalance, correction masses were added, and the resulting residual unbalances are presented in Table 7 and summarized by rotor type and correction plane in Table 8. The LSTM-based approach yields a lower residual unbalance than the average influence coefficient method in 13 of the 16 rotor–plane combinations, while the remaining three cases are unchanged and none deteriorates. For the three-slot rotors, the average residual unbalance decreased from 10.50 mg to 8.75 mg on correction plane I, a reduction of 16.7%, and from 9.25 mg to 7.75 mg on correction plane II, a reduction of 16.2%. For the five-slot rotors, the average residual unbalance decreased from 11.50 mg to 9.50 mg on plane I, a reduction of 17.4%, and from 9.25 mg to 8.25 mg on plane II, a reduction of 10.8%. Taken over all eight rotors, the reduction amounts to 17.0% on plane I and 13.5% on plane II, i.e., 15.4% of the total residual unbalance mass. For specific rotors on specific correction planes, the maximum reduction in residual unbalance mass reached 28.57%, demonstrating the superiority of the proposed LSTM-based method for micromotor rotor unbalance identification.

The three cases without reduction, rotor 3 of the three-slot type on correction plane II and rotors 1 and 2 of the five-slot type on planes II and I, share a common feature: the residual unbalance of both methods is already at the 6–8 mg level, corresponding to a specific unbalance of only 1.7–3.1 μm at the rotor radius of 23 mm. At such small residuals, the difference between the two methods lies below the resolution of the measurement chain itself. Also, the vibration response produced by a 6–8 mg residual is already close to the sensor noise floor. The absence of further improvement in these cases therefore reflects the resolution limit of the balancing process rather than a failure of the identification method.

The difference in identification effectiveness between the two methods is also reflected in the vibration amplitudes after balancing correction. Higher identification accuracy leads to smaller residual unbalance and consequently lower vibration amplitudes. Figure 8 compares the vibration responses of three-slot and five-slot rotors after balancing using the two methods. The response amplitudes obtained with the proposed method are substantially lower than those obtained with the traditional average influence coefficient method, confirming the effectiveness of the LSTM-based approach. Furthermore, the proposed method requires only a single constant-speed vibration measurement to identify the unbalance, eliminating the need for multiple start–stop cycles and trial-weight operations, thereby offering significantly higher detection efficiency than conventional approaches.

## 4. Conclusions

This paper has proposed an LSTM neural network-based method for identifying the unbalance mass and phase of micromotor rotors. The method uses a rotor-support dynamic model to generate synthetic vibration responses for pre-training, after which the network is fine-tuned with experimental measurements. The trained LSTM network maps the vibration amplitudes and phases at two measurement planes, together with key geometric parameters, directly to the unbalance masses and phases on the two correction planes.

The main contributions of this work are threefold. First, a dataset containing 10,081 simulated samples and 80 experimental samples was constructed, thereby mitigating the scarcity of labeled experimental data for micromotor rotors. Second, a multi-layer LSTM network was designed to directly predict the unbalance parameters on both correction planes, eliminating the need for trial weights and repeated start–stop cycles inherent in the conventional influence coefficient method. Third, the proposed method was experimentally validated on four three-slot and four five-slot micromotor rotors, demonstrating consistent improvements in balancing accuracy over the AIC method.

For the simulated data, the relative identification error was below 5% for unbalance masses above 40 mg, and the absolute error remained within 4 mg for smaller masses. For the experimental validation, the residual unbalance was reduced by 10.8–17.4% depending on rotor type and correction plane, corresponding to an overall reduction of 15.4% in total residual unbalance mass across the eight validation rotors, compared with the average influence coefficient method. Once trained, the proposed method requires only a single constant-speed vibration measurement per rotor, improving the balancing efficiency.

The present study is limited to three-slot and five-slot rigid micromotor rotors operating below the first critical speed under constant-speed conditions. Several additional limitations should be noted. First, the experimental validation involved only eight rotors with a typical unbalance range of micromotor production (20–150 mg). A larger and more diverse rotor batch would be needed to establish the statistical generality of the reported improvements. Second, training relies on a large amount of simulated data. However, in the current work, the simulated data involve only six variables, while the other parameters, including those related to the test rig, are held constant. This means that the boundary conditions are fixed, which limits the application of the model to other test environments. Third, the measurement uncertainty throughout the entire dynamic balancing process and its effect on the correction accuracy have not been quantified. Future work will focus on two directions: (i) deploying the trained LSTM model on an embedded measurement platform for online real-time balancing and (ii) extending the method to other rotor geometries, variable-speed operations, and flexible rotor systems operating above the first critical speed.

## Figures and Tables

**Table 1:** Network training parameters.

Parameter	Value
Solver	Adam
Batch Size	100
Epoch	100
Adam First-Moment Decay Factor	0.9
Initial Learn Rate	0.001
Learn Rate Drop Factor	0.2
Learn Rate Drop Period	200
Gradient Threshold	1
Validation Frequency	30

**Table 2:** Parameter descriptions.

Parameter	Abbreviation	Unit
Vibration amplitude at the left measurement plane	LA	μm
Vibration phase at the left measurement plane	LP	°
Vibration amplitude at the right measurement plane	RA	μm
Vibration phase at the right measurement plane	RP	°
Left correction unbalance mass	LUM	mg
Left correction unbalance phase	LUP	°
Right correction unbalance mass	RUM	mg
Right correction unbalance phase	RUP	°

**Table 3:** Input data samples.

Sample No.	*l*_1_	*l*_2_	LA	LP	RA	RP	LUM	LUP	RUM	RUP
1	28	30	9.30	247.5	2.81	270	61	137	121	219
2	28	30	2.22	303.75	6.78	33.75	88	204	26	73
3	30	30	3.11	0	14.56	157.5	175	164	17	98
4	34	31	3.68	11.25	9.43	123.75	117	83	35	292
5	32	27	7.48	202.5	7.88	112.5	94	64	102	113
6	27	26	5.88	270.0	7.60	270	111	30	85	118
7	26	28	8.82	258.75	14.37	135	160	334	111	87
8	29	32	3.50	146.25	15.76	11.25	188	154	43	171
9	31	33	14.28	112.5	6.51	33.75	87	216	189	234
10	32	34	6.08	123.75	12.65	258.75	151	200	57	265

**Table 4:** Input data after min–max normalization.

Sample No.	*l*_1_	*l*_2_	LA	LP	RA	RP	LUM	LUP	RUM	RUP
1	0.250	0.500	0.517	0.710	0.141	0.774	0.302	0.381	0.603	0.608
2	0.250	0.500	0.120	0.871	0.343	0.097	0.437	0.567	0.126	0.203
3	0.500	0.500	0.170	0	0.741	0.452	0.874	0.456	0.080	0.272
4	1	0.625	0.202	0.032	0.479	0.355	0.583	0.231	0.171	0.811
5	0.750	0.125	0.415	0.581	0.400	0.323	0.467	0.178	0.508	0.314
6	0.125	0	0.325	0.774	0.385	0.774	0.553	0.083	0.422	0.323
7	0	0.250	0.490	0.742	0.731	0.387	0.799	0.928	0.553	0.242
8	0.375	0.750	0.192	0.419	0.802	0.032	0.940	0.428	0.211	0.475
9	0.625	0.875	0.796	0.323	0.330	0.097	0.432	0.600	0.945	0.650
10	0.750	1	0.337	0.355	0.643	0.742	0.754	0.556	0.281	0.736

**Table 5:** Comparison of rotor unbalance identification results under simulated data.

Sample No.	Unbalance Parameter	Simulated Value	Identified Value	Relative Error (%)
1	LUM	61	59.2	−2.95
	LUP	137	142.2	3.80
	RUM	121	122.1	0.91
	RUP	219	212.4	−3.01
2	LUM	88	91.3	3.75
	LUP	204	203.3	−0.34
	RUM	26	22.0	−15.38
	RUP	73	69.1	−5.34
3	LUM	175	178.7	2.11
	LUP	164	157.7	−3.84
	RUM	17	20.2	18.82
	RUP	98	95.2	−2.86
4	LUM	117	120.5	2.99
	LUP	83	79.1	−4.70
	RUM	35	39.0	11.43
	RUP	292	286.0	−2.05
5	LUM	94	98.3	4.57
	LUP	64	60.0	−6.25
	RUM	102	102.9	0.88
	RUP	113	115.5	2.21

**Table 6:** Comparison of rotor unbalance identification results using the two methods.

Rotor Type	Rotor No.	AIC Method		LSTM Neural Network		Mass Deviation e*_m_* (%)	Phase Deviation e_φ_ (°)
		Correction Plane I	Correction Plane II	Correction Plane I	Correction Plane II		
Three-slot rotor	1	39 mg ∠ 182°	43 mg ∠ 294°	36 mg ∠ 174°	41 mg ∠ 288°	6.60%	7°
	2	78 mg ∠ 184°	25 mg ∠ 115°	76 mg ∠ 190°	28 mg ∠ 115°	6.65%	3°
	3	20 mg ∠ 47°	34 mg ∠ 329°	16 mg ∠ 43°	31 mg ∠ 338°	17.30%	7°
	4	29 mg ∠ 221°	65 mg ∠ 75°	33 mg ∠ 219°	66 mg ∠ 81°	6.80%	4°
Five-slot rotor	1	138 mg ∠ 99°	89 mg ∠ 80°	138 mg ∠ 94°	87 mg ∠ 77°	1.25%	4°
	2	97 mg ∠ 105°	90 mg ∠ 119°	98 mg ∠ 105°	88 mg ∠ 116°	1.65%	1.5°
	3	99 mg ∠ 149°	102 mg ∠ 120°	96 mg ∠ 156°	104 mg ∠ 121°	2.55%	4°
	4	150 mg ∠ 33°	41 mg ∠ 59°	151 mg ∠ 35°	44 mg ∠ 54°	3.75%	3.5°

**Table 7:** Comparison of residual unbalance after correction using the two methods.

Rotor Type	Rotor No.	AIC Method		LSTM Neural Network		Residual Unbalance Mass Reduction Rate of LSTM vs. AIC Method (%)	
		Correction Plane I	Correction Plane II	Correction Plane I	Correction Plane II	Correction Plane I	Correction Plane II
Three-slot rotor	1	10 mg ∠ 315°	11 mg ∠ 187°	9 mg ∠ 311°	9 mg ∠ 184°	10.00%	18.18%
	2	14 mg ∠ 40°	8 mg ∠ 225°	10 mg ∠ 42°	7 mg ∠ 217°	28.57%	12.50%
	3	7 mg ∠ 221°	6 mg ∠ 128°	6 mg ∠ 209°	6 mg ∠ 129°	14.29%	0%
	4	11 mg ∠ 352°	12 mg ∠ 193°	10 mg ∠ 344°	9 mg ∠ 189°	9.09%	25.00%
Five-slot rotor	1	12 mg ∠ 170°	8 mg ∠ 10°	10 mg ∠ 162°	8 mg ∠ 17°	16.67%	0%
	2	7 mg ∠ 160°	9 mg ∠ 9°	7 mg ∠ 156°	8 mg ∠ 355°	0%	11.11%
	3	12 mg ∠ 121°	12 mg ∠ 215°	10 mg ∠ 125°	10 mg ∠ 208°	16.67%	16.67%
	4	15 mg ∠ 102°	8 mg ∠ 331°	11 mg ∠ 96°	7 mg ∠ 342°	26.67%	12.50%

**Table 8:** Comparison of the residual unbalance achieved by the AIC and LSTM methods, by rotor type and correction plane.

Rotor Type	Correction Plane	Mean Residual, AIC (mg)	Mean Residual, LSTM (mg)	Reduction (%)	Cases Without Reduction
Three-slot	I	10.50	8.75	16.7	0 of 4
Three-slot	II	9.25	7.75	16.2	1 of 4 (rotor 3: 6 → 6 mg)
Five-slot	I	11.50	9.50	17.4	1 of 4 (rotor 2: 7 → 7 mg)
Five-slot	II	9.25	8.25	10.8	1 of 4 (rotor 1: 8 → 8 mg)
All eight rotors	I	11.00	9.12	17.0	1 of 8
All eight rotors	II	9.25	8.00	13.5	2 of 8
All eight rotors	Both planes	20.25	17.12	15.4	3 of 16

## Data Availability

The original contributions presented in this study are included in the article. Further inquiries can be directed to the corresponding author.

## Abbreviations

The following abbreviations are used in this manuscript:

LSTM	Long Short-Term Memory
ReLU	Rectified Linear Unit
RMSE	Root Mean Square Error
AIC	Average Influence Coefficient
DLI	Dynamic Load Identification
